# Rapid Development of Methicillin-Resistant *Staphylococcus aureus* (MRSA) Purulent Pericarditis in the Setting of Endocarditis

**DOI:** 10.1016/j.cjco.2021.06.020

**Published:** 2021-07-14

**Authors:** Samiullah Arshad, Naoki Misumida

**Affiliations:** a Department of Hospital Medicine, University of Kentucky, Lexington, Kentucky, USA; b Department of Cardiology, Gill Heart and Vascular Institute, University of Kentucky, Lexington, Kentucky, USA

## Abstract

Methicillin-resistant *Staphylococcus aureus* (MRSA) purulent pericarditis is a rare but potentially fatal complication of MRSA bacteremia. We describe a case of a 27-year-old patient with active intravenous drug use, who presented with fever, chills, and dyspnea and was found to have tricuspid valve endocarditis. Echocardiogram on admission showed no pericardial effusion. The patient became hypotensive, with worsening dyspnea, in the following 3 days. A computed tomography scan of the chest was repeated and showed a large pericardial effusion. The patient underwent pericardiocentesis and pericardial drain placement. Antibiotics were continued, with resolution of effusion. Early pericardiocentesis of a large purulent pericardial effusion may prevent catastrophic outcomes.

## Résumé

La péricardite purulente à *Staphylococcus aureus* résistant à la méthicilline (SARM) est une complication rare, mais potentiellement mortelle de la bactériémie à SARM. Nous décrivons le cas d’une patiente de 27 ans, consommatrice active de drogues par voie intraveineuse, qui présentait de la fièvre, des frissons et une dyspnée, et chez qui on a découvert une endocardite de la valve tricuspide. L’échocardiogramme à l’admission n’a montré aucun épanchement péricardique. La patiente est devenue hypotendue, et la dyspnée s’est aggravée, dans les trois jours suivants. Un tomodensitogramme de la poitrine a été répété, montrant un important épanchement péricardique. La patiente a subi une ponction péricardique et la pose d’un drain péricardique. Les antibiotiques ont été poursuivis, avec résolution de l’épanchement. La ponction péricardique précoce d’un important épanchement péricardique purulent peut prévenir des résultats catastrophiques.

Community-acquired methicillin-resistant *Staphylococcus aureus* (MRSA) infection is increasingly seen in patients who use intravenous drugs, who are 16 times more likely to develop an invasive infection than non–drug users.^1^ The proportion of invasive MRSA infections has increased from 4.1% in 2011 to 9.2% in 2016, per the Centers for Disease Control and Prevention.^1^ However, MRSA pericarditis is not commonly encountered in the current antibiotic era. Here, we report a case of purulent pericarditis that developed rapidly, in 3 days, despite the patient being on appropriate antibiotic therapy.

**Novel Teaching Points**

• MRSA purulent pericarditis may develop very rapidly in patients with MRSA bacteremia. Our report highlights the development of this complication in the span of 3 days.
• Our literature review suggests that the more common cause of MRSA purulent pericarditis is hematogenous seeding of the pericardium. However, patients should be screened for periannular complications if hemodynamic deterioration occurs.
• Early pericardiocentesis in a patient with stable hemodynamics should be considered to drain a large purulent pericardial effusion in the setting of MRSA endocarditis, to prevent tamponade, which may evolve very rapidly.

## Case

A 27-year-old woman with active intravenous drug use disorder presented with fever, cough, and shortness of breath. She reported the use of heroin 14 days prior to presentation. At presentation, her blood pressure was 100/70 mm Hg, heart rate 90 beats per minute, temperature 98.4°F, and oxygen saturation 91% on room air. Physical exam revealed basilar crackles on bilateral lung bases; cardiovascular exam was limited due to body habitus, but no murmur was identified on presentation.

### Initial investigations

**Figure 1:**
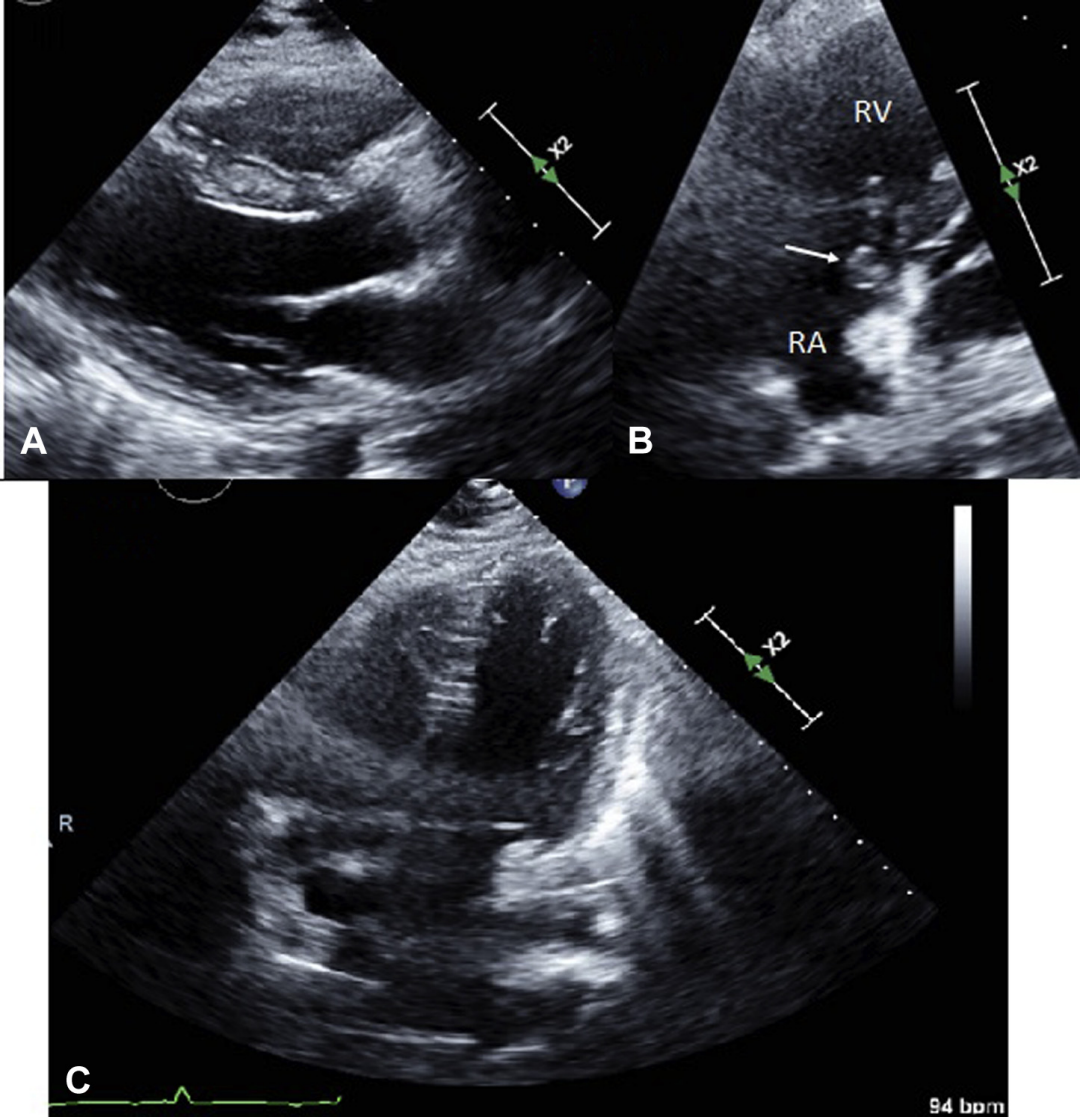
Echocardiogram upon admission. (**A**) Parasternal long-axis view revealed no pericardial effusion. (**B**) Right ventricular (RV) inflow view revealed a vegetation (**white arrow**) attached to tricuspid leaflet. (**C**) Apical 4-chamber view. RA, right atrium.

The initial white blood cell count was 21,200 per μL, and erythrocyte sedimentation rate was 95. Initial chest radiograph showed scattered cavitary nodules (Supplemental Fig. S1A). A computed tomography (CT) scan of the chest showed innumerable cavitary lung nodules scattered throughout the lungs consistent with septic emboli. Echocardiogram showed a mobile structure in the right atrium, consistent with vegetation on tricuspid leaflets with trace tricuspid regurgitation. No pericardial effusion was seen on admission (Fig. 1). Blood cultures were positive for MRSA.

### Management

**Figure 2:**
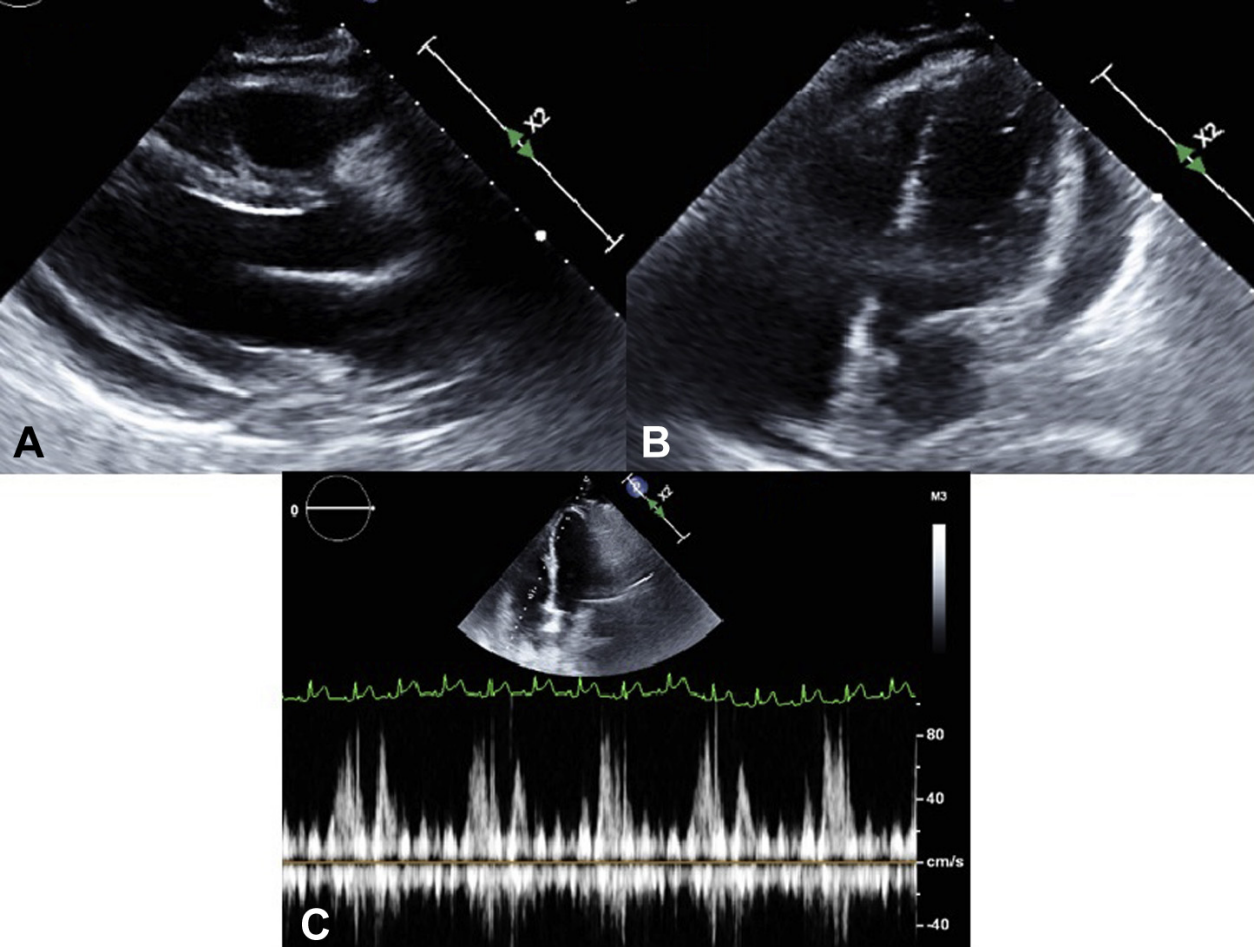
Echocardiogram on day 3. (**A**) Parasternal long-axis view revealed moderate-size circumferential pericardial effusion. (**B**) Apical 4-chamber view revealed moderate-size circumferential pericardial effusion. (**C**) Pulse-wave Doppler across the tricuspid valve revealed significant respiratory inflow variation. Every third cardiac cycle is associated with absent tricuspid inflow, presumably during expiration, with the patient breathing at a rate of 30/min, consistent with tamponade.

The patient was diagnosed with tricuspid valve infective endocarditis due to MRSA, complicated by multiple septic emboli. She was promptly started on intravenous vancomycin on presentation. Over the next 3 days, the patient continued to have worsening dyspnea. On day 3, the patient became hypotensive, with a blood pressure of 90/48 mm Hg, and tachycardic, with a heart rate of 115 beats per minute and new-oxygen requirement of 4 L supplemental oxygen via nasal cannula to maintain saturation above 88%. A chest radiograph showed worsening bilateral pulmonary infiltrates and a new right pleural effusion (Supplemental Fig. S1B). After fluid resuscitation, a repeat chest CT scan done for concern for lung abscess and empyema incidentally showed a moderate-to-large pericardial effusion (Supplemental Fig. S1C) and a moderate-size pleural effusion concerning for empyema. The patient did not have an obviously elevated jugular venous pressure, but assessment was difficult because of obesity. Pulsus paradoxus was not checked. A repeat echocardiogram showed moderate-size pericardial effusion with significant respiratory inflow variation across the tricuspid valve, inferior vena cava dilation but without chamber collapse, consistent with an early echocardiographic sign of cardiac tamponade (Fig. 2). No periannular extension of infection was seen on transthoracic echocardiogram. A transesophageal echocardiogram was not done. Tranthoracic echocardiogram and fluroscopy-guided pericardiocentesis was performed in the cardiac catheterization lab via a subxiphoid approach. A needle was inserted into the pericardium, and then a J-tipped wire was inserted into the pericardial space and confirmed by fluoroscopy. A dilator was advanced, and the wire was exchanged for a .035 J 260 cm Amplatz Super Stiff guide. An 8.3 F pigtail pericardial drain was then inserted over the wire and sutured into place. A total of 270 cc of bloody fluid was drained. Post-procedure blood pressure improved to 132/73 mm Hg. Pericardial fluid analysis revealed 892,500 per μL red blood cells, 3275 per μL nucleated cell, and a lactate dehydrogenase level of 1540 U/L. A pericardial fluid culture grew MRSA. Pericardial drain output stopped in 2 days, and the drain was removed 3 days after placement. A repeat echocardiogram showed an absence of pericardial fluid re-accumulation (Supplemental Fig. S2). Blood cultures became sterile within 5 days after presentation. Thoracentesis followed by chest tube placement for right pleural collection was completed 1 day after the pericardial drain placement, and pleural fluid analysis was consistent with empyema. Our plan was to continue intravenous vancomycin for 6 weeks, but the patient left the hospital against medical advice after having received 2 weeks of intravenous antibiotics.

## Discussion

Pericarditis is an inflammation of the pericardium, which can be associated with accumulation of pericardial fluid. Pericarditis is broadly categorized into infectious and noninfectious etiologies. Infectious causes include those that are viral, bacterial, fungal, and parasitic; noninfectious causes include those that neoplastic, auto-immune, metabolic, and drug-related.^2^

Bacterial pericarditis can be caused by hematogenous spread of bacteria, penetrating injury to the thoracic cavity, or direct spread from the surrounding structures, including the lungs, esophagus, and diaphragm, including the myocardium. Bacterial pericarditis is most commonly caused by *Staphylococcus*, *Streptococcus*, and tuberculous bacteria. Prior studies have reported *S. aureus* as the cause of pericarditis in 22% to 31% of cases.^3^

MRSA purulent pericarditis leading to tamponade is a rare but potentially fatal complication of MRSA bacteremia. A search of the literature on PubMed and Google Scholar for terms of MRSA pericarditis revealed 40 documented reports of MRSA pericarditis in adults, as summarized in Supplemental Table S1. Among these case reports, there were 47 patients, of which 5 patients (10%) used intravenous drugs. Cardiac tamponade was diagnosed in 29 patients (61%), and 31 patients (66%) underwent pericardiocentesis with drainage. Surgical intervention was done in 22 patients (47%), of whom 10 (21%) underwent pericardial window drainage, 5 (11%) underwent pericardiectomy, and 7 (15%) underwent concomitant mediastinal interventions for management of the infection.

Regueiro et al.^4^ reported that the prevalence of small-to-moderate pericardial effusions in the setting of infective endocarditis between 1990 and 2007 was 23%, and the prevalence of large pericardial effusions was 2%. Small-to-moderate pericardial effusions were more common in right-sided endocarditis in the setting of intravenous drug use. High circulating titers of immune complexes, a proinflammatory state, and immune response to infective endocarditis were proposed as causes of the effusion. Large pericardial effusions usually arise from periannular abscess with fistula formation and myocardial destruction extending to the pericardium. However, the study identified that large pericardial effusions also can be caused by hematogenous spread of *S. aureus* in the absence of periannular complications. A follow-up study by Regueiro et al.,^5^ using the Spanish Collaboration on endocarditis from 2007 to 2013, noted that 7.8% was mild-to-moderate pericardial effusion, and 0.5% was large pericardial effusion. Of 6 cases of large pericardial effusion, 2 were caused by *S. aureus*. Youssef et al.,^6^ in their study of 338 patients with endocarditis, highlighted that the presence of pericardial effusion in native or prosthetic valve endocarditis gave patients a worse prognosis, compared with that for patients without pericardial effusion. Further studies are needed to quantify the proportion of purulent vs serous collections in moderate and large pericardial effusions in the setting of endocarditis.

A review of the literature on cases of MRSA pericarditis revealed that only 1 patient had endomyocardial extension of the infection (Tani et al. in Supplemental Table S1) leading to pericardial effusion, with the remaining cases with likely hematogenous seeding of the pericardium leading to pericarditis. Hematogenous seeding was likely the mechanism of pericardial effusion in our patient. Another possible mechanism for the development of pericarditis in our patient is the contiguous spread of infection from the lungs in the setting of septic emboli and empyema.

Clinical features of purulent pericarditis, including classic positional chest pain, pericardial friction rub, and electrical alternans, may be absent in the initial few days. An echocardiogram is the most sensitive way to identify pericardial effusion; significant pericardial effusion can also be identified on a CT chest scan, as in our case. Typical echocardiographic signs of tamponade, including prominent right atrial and right ventricular collapse in the setting of clinical tamponade, may be absent in severe pulmonary hypertension and severe right ventricular hypertrophy.^7^ Respiratory septal motion may be attenuated in the setting of a rigid hypertrophied septum. Our patient had subtle echocardiographic signs of early tamponade, and lacked the right atrial/right ventricular collapse, without any of the above conditions masking them. Tamponade is not an all-or-none phenomenon; hence, both clinical and early echocardiographic findings should be taken into consideration for decision-making about intervention. The rapid improvement in hemodynamics post-pericardiocentesis supports the diagnosis of cardiac tamponade. More than half of patients with MRSA pericarditis developed cardiac tamponade, and about half of them required surgical drainage. Hence, for a large pericardial effusion in the setting of endocarditis, we recommend early intervention to prevent sudden hemodynamic compensation, as the purulent fluid may rapidly collect in the pericardial space.

Pericardial fluid analysis usually reveals a low glucose level and increased white and red blood cell counts, with an elevated lactate dehydrogenase level indicative of exudative pericardial effusion. The rapid development and progression of purulent effusion to cardiac tamponade is near fatal in 85% of cases if left untreated.^8^

Management includes antibiotics and drainage. Drainage usually includes pericardial drain placement with control of bacteremia. Intrapericardial streptokinase may help dissolve the septations and improve drainage. Surgical interventions include pericardiotomy and pericardial window placement, which may be needed for recurrent accumulations.

## Conclusion

Our case and review of the literature highlight the fact that patients with MRSA endocarditis can rapidly develop a large purulent pericardial effusion, and early pericardiocentesis may prevent a catastrophic outcome.
