# Supplementary material for: Rapid Development of Methicillin-Resistant Staphylococcus aureus (MRSA) Purulent Pericarditis in the Setting of Endocarditis
Source: CJC Open. 2021 Jul 14;3(12):1505–8. doi: 10.1016/j.cjco.2021.06.020 (PMC8712604; doi:10.1016/j.cjco.2021.06.020)
Supplement: Supplementary Material [file mmc1.pdf]

Supplementary Material

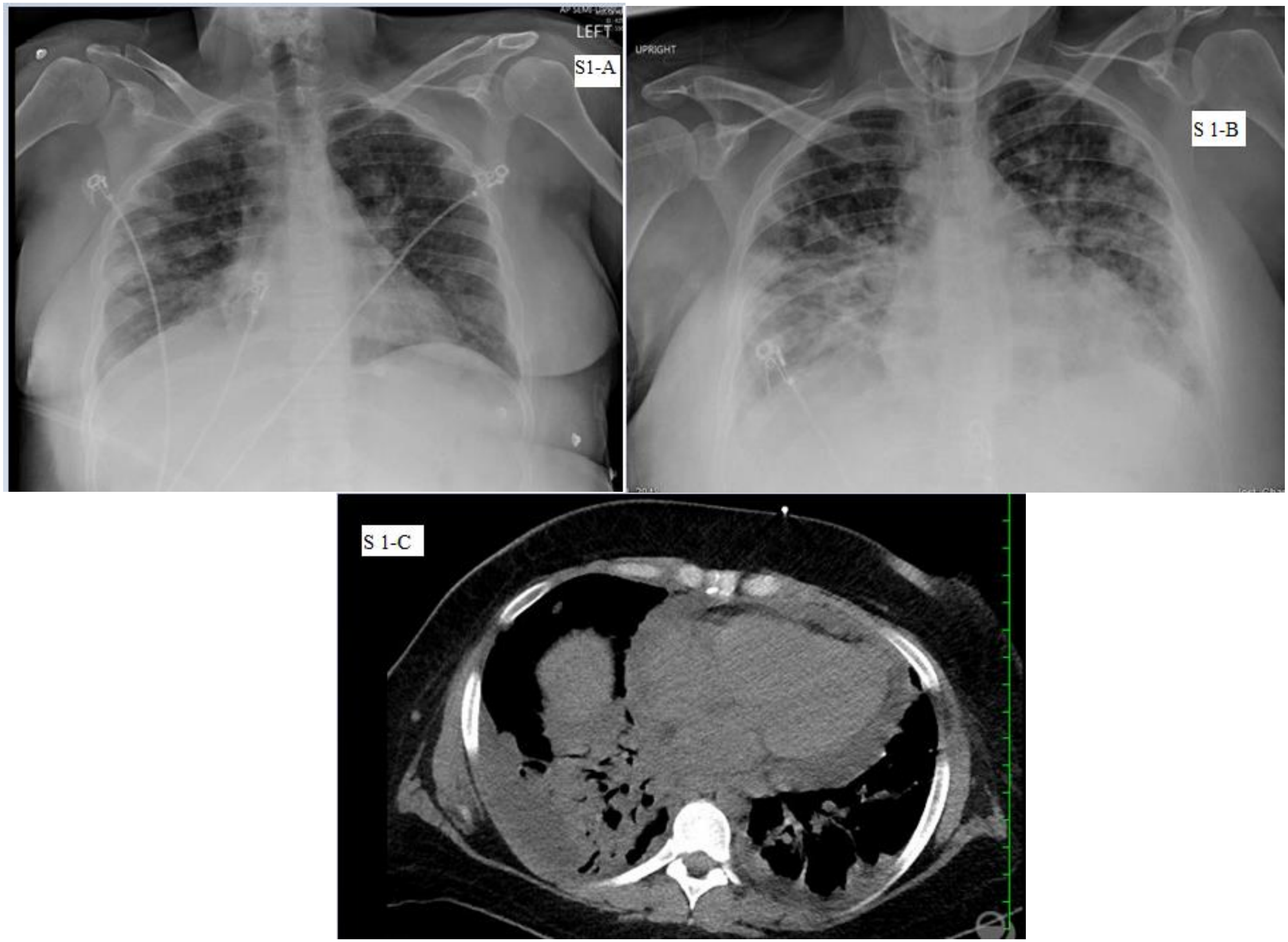

Supplemental Figure S1: Chest Imaging: S1-A Chest Xray on presentation with bilateral infiltrates without significant right pleural effusion, S1-B Chest Xray 3 days into hospitalization with a right pleural effusion and worsening pulmonary infiltrates. S1-C CT Chest done to assess for the right pleural effusion, with a moderate to large pericardial effusion.

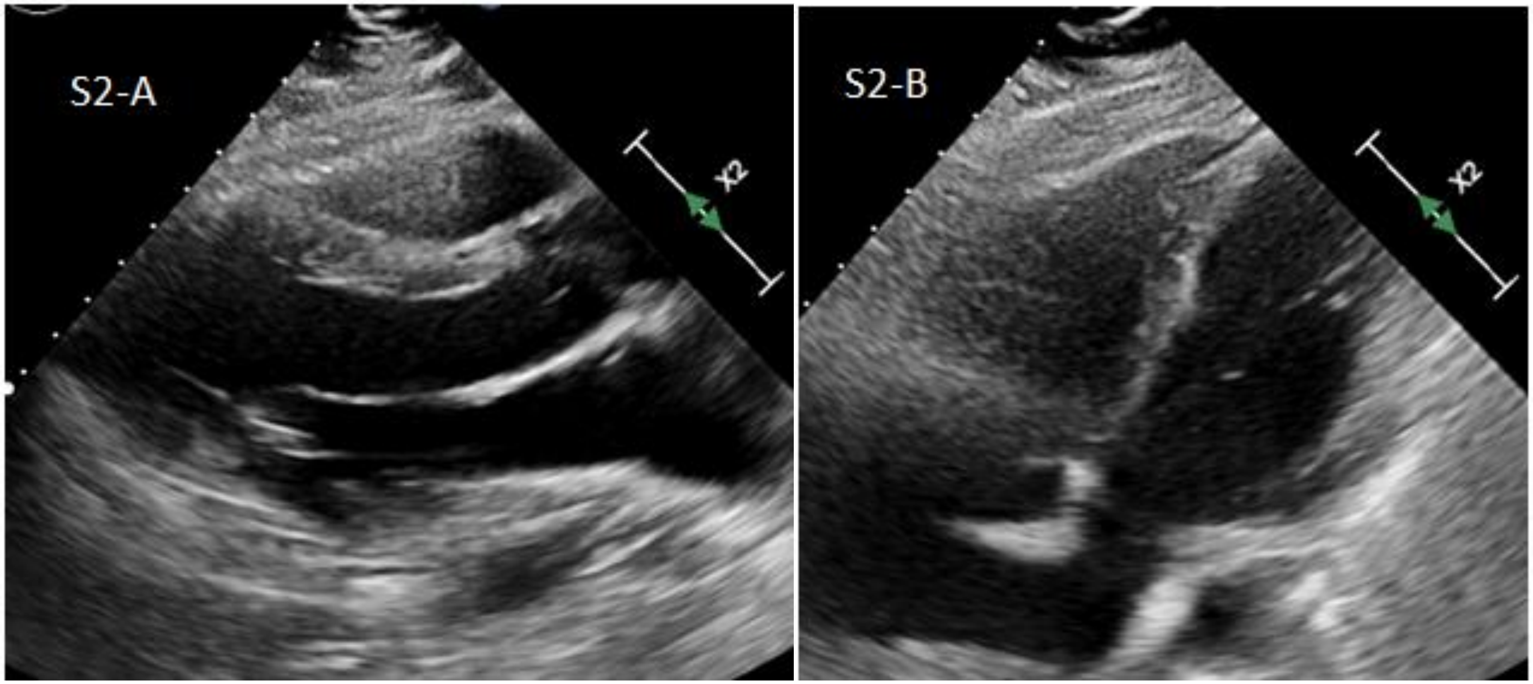

Supplemental Figure S2: Echocardiogram on day 6 post pericardiocentesis. S2-A; Parasternal long axis view revealed no residual pericardial effusion. 2-B; Apical four-chamber view revealed no residual pericardial effusion.

Supplemental Table S1: Summary of reported cases of MRSA pericarditis described in adults. (ESRD- End Stage Renal Disease, IVDU- Intravenous Drug Use)

Authors	Age, Gender	Predisposing conditions	Tamponade	Pericardiocentesis with drainage	Surgical Intervention	Medical management and Outcomes
Parikh et al (1)	54, Male	Splenectomy	Yes	Yes	Pericardial Window	Completed 6 weeks antibiotics
Kariyanna et al (2)	54, Female	Esophago-Pericardial fistula in setting of invasive SCC	Yes	No	Pericardial Window	Died, due to septic shock.
DeYoung et al (3)	48, Male	Elbow abscess	Yes	Yes	Pericardiectomy	complicated by Drug Reaction with Eosinophilia and Systemic Symptoms (DRESS)
Mada et al (4)	36, Female	DRESS	Yes	Yes	Pericardiectomy	Pericardiectomy done after sepsis subsided
Shihadeh et al (5)	29, Female	Diabetes Mellitus Type 2, Scalp abscess In setting of hair implants.	Yes	Yes	Pericardiectomy	Pericardiectomy done, patient died due to septic shock
Dherange et al (6)	55, Female	Diabetes Mellitus Type 2, ESRD, Foot osteomyelitis	No	Yes	No	Successful treatment with 6 weeks of antibiotics, below knee amputation
Gunatilake et al (7)	45, Female	Lemierre's syndrome	Yes	Yes(multiple)	No	Completion of antibiotics for 6 weeks.
Kumar et al (8)	78, Male	SCC of buccal mucosa, Renal Failure	Yes	Yes	No	Died due to renal failure and septic shock
Belton et al (9)	38, Female	MRSA bacteremia with pericarditis due to Bath Salt injections	Not described	Not Described	Not described	Left against medical advice after 15 days of antibiotics
Hussam et al	19, Male	None identified	Yes	Not described	Pericardial	antibiotics completed for 6

(10)					window	weeks
Saito et al (11)	66, Female	Recent surgery	Yes	Yes	Pericardial window via Subxiphoid approach	Had concomitant infected aortic aneurysm, died despite surgical intervention.
Lee et al (12)	41, Male	MRSA Pneumonia	No	No	No	Myopericarditis improved after antibiotics
Patel et al (13)	20, Male	Lower extremity skin infection	Yes	Yes	Surgical drainage with pseudoaneurysm repair	Infected aortic pseudoaneurysm, surgical management and completion of antibiotics
Abiko et al (14)	72, Female	Esophageal perforation, small cell lung carcinoma	Not described	Not described	Pericardial and Mediastinal drainage	Died
Muralikrishnan et al (15)	Ages and gender not described 5 patients	Surgical patients	Not described	Not Described	Not described	All 5 patients Died
Evron et al (16)	73, Male	Pulmonary Lymphoma	Not described	Not described	Not described	Completed antibiotic therapy
Kurahara et al (17)	60, Male	Non-small cell Lung cancer	Yes	Yes	No	Improvement after antibiotic therapy
Oizumi et al (18)	58, Male	ESRD	Yes	Yes	Pericardiotomy with pericardial window	discharged with antibiotics
Chawla et al (19)	25, Male	IVDU	Yes	Yes	Surgical Pericardial drainage	Not described
Ibrahim et al (20)	31, Female	IVDU	Yes	Yes	Pericardial window	Not described
Saffarian et al (21)	32, Male	IVDU	No	Yes	No	6 weeks of antibiotics after pericardial drainage

Holden et al (22)	49, Male	Cirrhosis	Yes	Yes	Pericardial Window	Antibiotics completed for 4 weeks
Ali et al (23)	46, Female	HIV/AIDS	Yes	Yes	No	Deemed not a surgical candidate, was managed with antibiotics, aspirin and colchicine
Meena et al (24)	18, Male	None	Yes	Yes	No	Managed with IV antibiotics
Arora et al (25)	58, Female	Diabetes Mellitus, Cocaine use	Yes	Yes	No	Managed with antibiotics
Terada et al (26)	75, Female	Lung cancer with recent chemotherapy	Yes	Yes	Subxiphoid pericardial drainage	Daily intrapericardial saline wash, in addition to antibiotics used
Ganji et al (27)	66, Female	Diabetes Mellitus Type 2, history of breast cancer, recent Influenza A infection	Yes	No	Pericardial Window	Antibiotics and colchicine
Kondapi et al (28)	68, Female	None	Yes	Yes	No	Drainage and 6 weeks of antibiotics
Tan et al (29)	44, Male	Diabetic foot ulcer	Yes	Yes	No	Died despite antibiotic therapy
Shivaraju et al (30)	34, Female	IVDU	Yes	Yes	No	6 weeks of antibiotics after pericardial drainage
Ma et al (31)	48, Male	Cellulitis	Yes	Yes	Pericardiectomy	Persistent Myocarditis and developed heart failure
Patel et al (32)	60, Male	Coronary Stent Infection	Yes	Yes	Coronary Artery Bypass Graft	Antibiotics initially, CABG done later for mycotic aneurysm of Left Anterior descending coronary artery
Zarour et al (33)	42, Male	ESRD	Yes	Yes	No	Treated with antibiotics
Unkown authors (34)	31, Male	Immunosuppression	Unknown	Yes	No	Died

Rao et al (35)	68, Female	Prior MRSA infection	No	No	Pericardiectomy	6 weeks of antibiotics with improvement
Bryne et al (36)	38, Female	None	Yes	Yes	Pericardial Window	Not described
Tani et al (37)	28, Female	Infective endocarditis extending to pericardium	No	No	Surgical drainage with Mitral valve repair, left atrial reconstruction and annuloplasty	Completed 6 weeks of antibiotics
Khutan et al (38)	17, Male	Soft tissue infection of leg	Yes	Yes	No	Completed 6 weeks of antibiotics
Redwood et al (39)	**4 cases**					
	41, Female	Skin	Yes	Yes	No	Completed Antibiotic therapy
	32, Female	Popping of heroine	No	No	Pericardial window	Diffuse fibrosing pericarditis, completed antibiotics
	31, Female	Infected Port	No	No	Purulent abscess drainage	Completed antibiotic therapy
	52, Female	Cellulitis, Diabetes Mellitus Type 2 Diabetes Mellitus type 2, Skin Popping	Not described	Yes	No	Improvement after pericardiocentesis
Kang et al (40)	41, Male	ESRD, Gastric Cancer	No	Yes	No	Completed 6 weeks of antibiotics

References for Supplementary Table S1:

1. Parikh SV, Memon N, Echols M, Shah J, McGuire DK, Keeley EC. Purulent pericarditis: report of 2 cases and review of the literature. Medicine (Baltimore). 2009 Jan;88(1):52-65. doi: 10.1097/MD.0b013e318194432b. PMID: 19352300.
2. Kariyanna PT, Jayarangaiah A, Pedalino R, Hegde S, Marmur JD, Shenoy A, Ashamalla M, Ray J, McFarlane SI. Esophago-pericardial Fistula Induced Community Acquired Methicillin Resistant Staphylococcus Aureus (CA-MRSA) Cardiac Tamponade - A Rare Case Report and Literature Review. Am J Med Case Rep. 2018;6(6):109-113. doi: 10.12691/ajmcr-6-6-3. Epub 2018 Jul 10
3. DeYoung H, Bloom A, Tamayo S. Successful treatment of community-acquired methicillin-resistant Staphylococcus aureus purulent myopericarditis. BMJ Case Rep. 2017 Oct 10;2017:bcr2017221931. doi: 10.1136/bcr-2017-221931.
4. Mada PK, Cady B, De Silva A, Alam M. Disseminated MRSA infection with purulent pericarditis. BMJ Case Rep. 2017 Mar 30;2017:bcr2016218463. doi: 10.1136/bcr-2016-218463
5. Anmad Shihadeh L, Couto Comba P, Hernández Carballo C. Purulent pericarditis by Staphylococcus Panton-Valentine secondary to hair implants. Med Clin (Barc). 2017 Jun 7;148(11):525. English, Spanish. doi: 10.1016/j.medcli.2017.02.003
6. Dherange PA, Patel S, Enakpene E, Suryanarayana P. From bone to heart: a case of MRSA osteomyelitis with haematogenous spread to the pericardium. BMJ Case Rep. 2015 Dec 7;2015:bcr2015211410. doi: 10.1136/bcr-2015-211410. PMID: 26643184; PMCID: PMC4680271.
7. Gunatilake SS, Yapa LG, Gallala M, Gamlath R, Rodrigo C, Wimalaratna H. Lemierre's syndrome secondary to community-acquired methicillin-resistant Staphylococcus aureus infection presenting with cardiac tamponade, a rare disease with a life-threatening presentation: a case report. Int J Emerg Med. 2014 Sep 26;7:39. doi: 10.1186/s12245-014-0039-y. PMID: 25635199; PMCID: PMC4306077.
8. Kumar VA, Nair N, Thachathodiyl R, Nandakumar A, Dinesh KR, Thatcher E, Karim S, Biswas R. Molecular Characterization of Methicillin-Resistant Staphylococcus aureus Causing Fatal Purulent Pericarditis. J Lab Physicians. 2013 Jul;5(2):136-8. doi: 10.4103/0974-2727.119874. PMID: 24701111; PMCID: PMC3968627.
9. Belton P, Sharngoe T, Maguire FM, Polhemus M. Cardiac infection and sepsis in 3 intravenous bath salts drug users. Clin Infect Dis. 2013 Jun;56(11):e102-4. doi: 10.1093/cid/cit095. Epub 2013 Feb 15. PMID: 23418275.
10. Hussam MA, Ragai MF, Iman MF, Zakaria A. Community-acquired methicillin-resistant Staphylococcus aureus pericarditis presenting as cardiac tamponade. South Med J. 2010 Aug;103(8):834-6. doi: 10.1097/SMJ.0b013e3181e631e7. PMID: 20622739.
11. Saito S, Matsuura A, Miyahara K, Takemura H, Sawaki S, Ito H. Infected aortic aneurysm, purulent pericarditis, and pulmonary trunk rupture caused by methicillin-resistant Staphylococcus aureus. Gen Thorac Cardiovasc Surg. 2009 May;57(5):250-2. doi: 10.1007/s11748-008-0376-y. Epub 2009 May 15. PMID: 19440821.
12. Lee YP, Hoi WH, Wong RC. A case of myopericarditis in a patient with methicillin-resistant Staphylococcus aureus community-acquired pneumonia. Ann Acad Med Singap. 2008 Mar;37(3):243-2. PMID: 18392307.

13. Patel S, Maves R, Barrozo CP, Mullens F, Russell K, Truett A, Deroo T. Mycotic pseudoaneurysm and purulent pericarditis attributable to methicillin-resistant Staphylococcus aureus. Mil Med. 2006 Aug;171(8):784-7. doi: 10.7205/milmed.171.8.784. PMID: 16933824.
14. Abiko M, Ohizumi H, Naruke Y, Takeda F, Koshika M, Yuki Y, Shimazaki Y. [A case of lung cancer (small cell carcinoma) occurring esophago-pericardial fistula and purulent pericarditis]. Kyobu Geka. 1999 Oct;52(11):969-71. Japanese. PMID: 10513169.
15. Muralikrishnan VP, Thomas DH, Elbouri K. The association of methicillin resistant Staphylococcus aureus and pericarditis. J Hosp Infect. 1998 Dec;40(4):328. doi: 10.1016/s0195-6701(98)90314-0. PMID: 9868628.
16. Evron E, Goland S, Somin M, Sthoeger ZM. [Purulent pericarditis]. Harefuah. 1996 May 1;130(9):602-3, 655. Hebrew. PMID: 8794638.
17. Kurahara Y, Kawaguchi T. Cardiac tamponade with community-acquired methicillin-resistant Staphylococcus aureus pericarditis. Intern Med. 2013;52(15):1753. doi: 10.2169/internalmedicine.52.0542. Epub 2012 Mar 1. PMID: 23903515
18. Oizumi H, Ichinokawa H, Hoshino H, Shitara J, Suzuki K. Pericardial Window for Methicillin-Resistant Staphylococcus aureus Pericarditis. Ann Thorac Surg. 2019 Jan;107(1):e27-e29. doi: 10.1016/j.athoracsur.2018.05.053. Epub 2018 Jun 19. PMID: 29932888.
19. Chawla L, Katchi T, Koulova A, Kabra N, Gupta S, Yandrapalli S, et al. METHICILLIN RESISTANT STAPHYLOCOCCUS AUREUS CAUSING PURULENT PERICARDITIS IN THE SETTING OF INTRAVENOUS DRUG USE. J Am Coll Cardiol 2018;71:A2337. https://doi.org/10.1016/S0735-1097(18)32878-X.
20. Ibrahim AM, Bhandari B, Gudivada S, Mahmaljy H, Kulkarni A. PURULENT PERICARDITIS DUE TO METHICILLIN-RESISTANT STAPHYLOCOCCUS AUREUS COMPLICATED BY CARDIAC TAMPONADE. J Am Coll Cardiol 2019;73:2753. https://doi.org/10.1016/S0735-1097(19)33359-5.
21. Saffarian M, Xu PZ, Mehra A, Ostrzega E. UNUSUAL SURVIVAL IN A PATIENT WITH MRSA PERICARDITIS WITH RAPIDLY PROGRESSIVE PERICARDIAL EFFUSION AND ABSCESS TREATED MEDICALLY IN COMBINATION WITH PERCUTANEOUS INTERVENTION. J Am Coll Cardiol 2020;75:3159. https://doi.org/10.1016/S0735-1097(20)33786-4.
22. Holden K, Hussain M, Brownell B. Pus in the Pericardium: A Case of MRSA Pericarditis. D47 CRITICAL CARE CASE REPORTS: INFECTION AND SEPSIS II:A6965-A6965.
23. Ali L, Ghazzal A, Sallam T, Cuneo B. Rapidly Developing Methicillin-Resistant Staphylococcus Aureus Pericarditis and Pericardial Tamponade. Cureus. 2020 May 7;12(5):e8001. doi: 10.7759/cureus.8001. PMID: 32528743; PMCID: PMC7279682.
24. Meena DS, Kumar D, Gopalakrishnan M, Bohra GK, Midha N, Vijayvargiya P, Tiwari S. Purulent pericarditis in a patient with community-acquired methicillin-resistant *Staphylococcus aureus*: a case report with mini-review. Germs. 2020 Sep 1;10(4):249-253. doi: 10.18683/germs.2020.1212. PMID: 33134204; PMCID: PMC7572210
25. Arora NP, Kottam A, Mahajan N, Bhasin B, Krishnamoorthi R, Shenoy M, Afonso LC. Purulent pericardial effusion from community-acquired methicillin-resistant Staphylococcus aureus. Am J Med Sci. 2012 Aug;344(2):160-2. doi: 10.1097/MAJ.0b013e31824e942b. PMID: 22627263.
26. Terada M, Watanabe H, Kobukai Y, et al. Successful treatment of a patient with purulent pericarditis by daily intrapericardial washouts. Ann Thorac Surg. 2014;98:1451–4. doi: 10.1016/j.athoracsur.2013.11.072.

27. Ganji M, Ruiz J, Kogler W, Lung J, Hernandez J, Isache C. Methicillin-resistant *Staphylococcus aureus* pericarditis causing cardiac tamponade. IDCases. 2019 Aug 1;18:e00613. doi: 10.1016/j.idcr.2019.e00613. PMID: 31453103; PMCID: PMC6704044.
28. Kondapi D, Markabawi D, Chu A, Gambhir HS, "Staphylococcal Pericarditis Causing Pericardial Tamponade and Concurrent Empyema", *Case Reports in Infectious Diseases*, vol. 2019, Article ID 3701576, 5 pages, 2019
29. Tan TL, Lim SH, Ruslan Mustapa M, Ganesway R. Pericardial abscess: The corollary of disseminated Methicillin-resistant Staphylococcus aureus following diabetic foot ulcer infection. Med J Malaysia. 2020 Nov;75(6):742-744. PMID: 33219189.
30. Shivaraju A , Luu J , Bratis C , Akhter N , Stamos T: **Cardiology Review Online**, January 2008, Volume 26, Issue 1
31. Lucy Ma, BS, Jed Mangal, MD, Vincent Capaldi, MD, From Skin Infection to Pericardiectomy: A Cautionary Tale of Undertreated Methicillin-Resistant *Staphylococcus aureus*, *Open Forum Infectious Diseases*, Volume 3, Issue suppl_1, December 2016, 1061, https://doi.org/10.1093/ofid/ofw172.764
32. Patel AJ, Mehta RM, Gandhi DB, Bossone E, Mehta RH. Coronary aneurysm and purulent pericardial effusion: old disease with an unusual cause. Ann Thorac Surg. 2013 May;95(5):1791-3. doi: 10.1016/j.athoracsur.2012.09.045. PMID: 23608264.
33. Zarour CC, Dervishi M, Fuguet D, Al-bahbahanee Z. Methicillin-Resistant Staphylococcus aureus (MRSA)-Positive Pericardial Abscess Presenting in a Hemodialysis Patient. Cureus. 2020 Sep 12;12(9):e10411. doi: 10.7759/cureus.10411. PMCID: PMC7550242.
34. Immunosuppressants. React Wkly 2009;1251:22. https://doi.org/10.2165/00128415-200912510-00064.
35. Rao, S.K., Protopapas, Z. & Vikram, H.R. Unusual presentation of purulent pericarditis: diagnostic contribution of MRI. *Emergency Radiology* **10,** 152–154 (2003).
36. Byrne R, Bradham W. SPONTANEOUS PURULENT PERICARDITIS DUE TO METHICILLIN-RESISTANT STAPHYLOCOCCUS AUREUS: A CASE REPORT AND DISCUSSION. J Am Coll Cardiol 2017;69:2112. https://doi.org/10.1016/S0735-1097(17)35501-8.
37. Tani, T., Okada, Y., Kita, T. *et al.* Destructive acute infective endocarditis and purulent pericarditis. *J Echocardiogr* **11,** 164–166 (2013).
38. Khutan H, Kaur R, Singh G, Singh P, Kaur A. Case of disseminated community-acquired methicillin-resistant *Staphylococcus aureus*: Unique behavior of the organism in an immunocompetent adult. Arch Med Health Sci 2018;6:254-6
39. Redwood, Marcia MD; McCabe, Robert MD*† Four Cases of Community-Associated Methicillin-Resistant Staphylococcus aureus Pericarditis, Infectious Diseases in Clinical Practice: July 2010 - Volume 18 - Issue 4 - p 251-252 doi: 10.1097/IPC.0b013e3181d5ab68
40. Kang, Hye & Kim, So & Lee, Eun & Kim, Tae & Park, byoung won. (2014). Primary Methicillin-Resistant Staphylococcus aureus Pericarditis in a Patient Undergoing Hemodialysis. Soonchunhyang Medical Science. 20. 42-44. 10.15746/sms.14.010.
